# Determinants of the VP1/2A junction cleavage by the 3C protease in foot-and-mouth disease virus-infected cells

**DOI:** 10.1099/jgv.0.000664

**Published:** 2017-04-01

**Authors:** Thea Kristensen, Preben Normann, Maria Gullberg, Ulrik Fahnøe, Charlotta Polacek, Thomas Bruun Rasmussen, Graham J Belsham

**Affiliations:** National Veterinary Institute, Technical University of Denmark, Lindholm, DK-4771 Kalvehave, Denmark; ^†^​ Present address: Copenhagen Hepatitis C Program (CO-HEP), Department of Infectious Diseases and Clinical Research Centre, Hvidovre Hospital and Department of International Health, Immunology and Microbiology, Faculty of Health and Medical Sciences, University of Copenhagen, Denmark.

**Keywords:** picornavirus, polyprotein processing, cleavage specificity, virus capsid assembly, proteolysis

## Abstract

The foot-and-mouth disease virus (FMDV) capsid precursor, P1-2A, is cleaved by FMDV 3C protease to yield VP0, VP3, VP1 and 2A. Cleavage of the VP1/2A junction is the slowest. Serotype O FMDVs with uncleaved VP1-2A (having a K210E substitution in VP1; at position P2 in cleavage site) have been described previously and acquired a second site substitution (VP1 E83K) during virus rescue. Furthermore, introduction of the VP1 E83K substitution alone generated a second site change at the VP1/2A junction (2A L2P, position P2′ in cleavage site). These virus adaptations have now been analysed using next-generation sequencing to determine sub-consensus level changes in the virus; this revealed other variants within the E83K mutant virus population that changed residue VP1 K210. The construction of serotype A viruses with a blocked VP1/2A cleavage site (containing K210E) has now been achieved. A collection of alternative amino acid substitutions was made at this site, and the properties of the mutant viruses were determined. Only the presence of a positively charged residue at position P2 in the cleavage site permitted efficient cleavage of the VP1/2A junction, consistent with analyses of diverse FMDV genome sequences. Interestingly, in contrast to the serotype O virus results, no second site mutations occurred within the VP1 coding region of serotype A viruses with the blocked VP1/2A cleavage site. However, some of these viruses acquired changes in the 2C protein that is involved in enterovirus morphogenesis. These results have implications for the testing of potential antiviral agents targeting the FMDV 3C protease.

## Introduction

*Foot-and-mouth disease virus* (FMDV) is the prototypic member of the genus *Aphthovirus* within the family *Picornaviridae* and seven different serotypes (O, A, C, SAT 1–3 and Asia-1) have been identified. All FMDVs have a positive-sense RNA genome (ca. 8400 nt) that includes a single large ORF (ca. 7000 nt) encoding a polyprotein [1]. The full-length polyprotein is never observed since during, and after, synthesis it is processed, mainly by virus-encoded proteases, to generate 15 distinct mature products plus multiple precursors. The FMDV polyprotein includes two *trans*-acting proteases; these are the Leader (L) protease and the 3C protease (3C^pro^). The L protease is only responsible for one cleavage within the polyprotein that occurs at its own C-terminus (i.e. the L/P1-2A junction, [2, 3]). However, this protease also induces cleavage of the translation initiation factor eIF4G; this results in the inhibition of host cell, cap-dependent, protein synthesis (reviewed in [1]). The 3C^pro^ cleaves all the other junctions within the FMDV polyprotein except for the VP4/VP2 junction and the 2A/2B junction. Cleavage of VP0 to VP4 and VP2 occurs on encapsidation of the viral RNA and also within assembled empty capsid particles [4–6]. Separation of the 2A peptide from the 2B protein is dependent on the 2A coding sequence. However, this region only encodes 18 aa (without any protease motifs), but its presence results in a break in the polyprotein during its synthesis; this is described as ‘ribosomal skipping’ [7] or ‘StopGo’ [8].

**Fig. 1.**
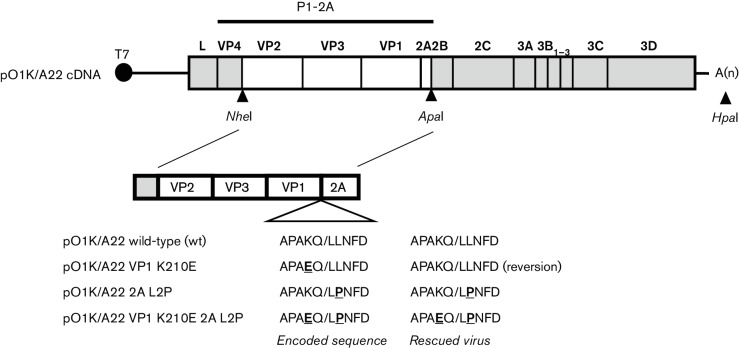
Schematic structure of the plasmid containing the FMDV O1 Kaufbeuren (O1K)/A22 cDNA and derivatives. The *Nhe*I and *Apa*I restriction enzyme sites (as indicated) were used as described in Methods to introduce cDNA fragments encoding the serotype A FMDV capsid proteins VP2-VP3-VP1-2A (from A22 Iraq, white fill) into the plasmid pT7S3 [33], containing a full-length cDNA corresponding to the O1K B64 strain of FMDV (coding sequences marked in grey). The plasmid-encoded amino acid sequences at the VP1/2A junction are shown. The FMDV O1K/A22 wt, single mutants (VP1 K210E or 2A L2P) and double mutant (VP1 K210E and 2A L2P) were produced as described in Methods. The full-length plasmids were linearized using *Hpa*I prior to *in vitro* transcription and virus rescue. The locations of restriction sites used are marked: *Nhe*I, *Apa*I and *Hpa*I. Sequence changes in the capsid coding region of the rescued viruses are indicated.

The FMDV capsid protein precursor, P1-2A (Fig. 1), is processed by the 3C^pro^ to VP0, VP3, VP1 and 2A. The scission of the VP1-2A junction is the slowest of these cleavages within cell-free translation systems [9] and within mammalian cells [5, 6] since the uncleaved VP1-2A can still be detected when all other junctions are fully cleaved (e.g. when P1-2A is expressed with a low level of 3C^pro^). However, in peptide cleavage assays, using short synthetic substrates, it has been found that the peptide corresponding to the VP1/2A cleavage site was the most rapidly processed [10].

The FMDV 3C^pro^ cleaves a variety of different junction sequences (the amino acid residues at the cleavage junctions are indicated as P4P3P2P1/P1′P2′P3′P4′). The cleavage sites recognized by the FMDV 3C^pro^ have either glutamine (Gln, Q) or glutamate (Glu, E) at the P1 position [11]. The consensus sequence (in single-letter code) for the VP1/2A junction in serotype O and A FMDVs is PxKQ/xLNF. The Q residue at the P1 position, together with the P4-Pro (P), P2-Lys (K) and P4′-Phe (F) residues, represent key determinants of 3C^pro^ specificity at this site [10–12]. Analysis of aligned FMDV 3C^pro^ cleavage sites from over 100 strains of the virus (including representatives of all serotypes) revealed that sites with P1-Q have a strong selectivity for P2-K, indicating that recognition of the P1 residue by 3C^pro^ is influenced by the P2 residue [11]. Recently, we have shown that changing the P2-K residue to E at the VP1/2A junction (i.e. K210E in VP1), in a serotype O virus, greatly inhibited cleavage at this junction and resulted in the formation of infectious virus particles containing the uncleaved VP1-2A [6]. The ‘self-tagged’ viruses containing this modification (K210E) also acquired, during the virus rescue procedure, a second amino acid substitution within VP1 (E83K). Interestingly, introduction of this E83K substitution alone into the virus generated a second site mutant (L2P, in the 2A sequence; this is position P2′ in the VP1/2A junction) that also blocked cleavage [13]. We have now expanded this analysis to identify the determinants of cleavage at the VP1/2A junction within the context of infectious serotype O and A FMDVs using a variety of different approaches. Within the serotype A FMDVs, no second site changes in VP1 were observed in viruses where the VP1/2A cleavage was inhibited, but some evidence for changes in 2C was obtained. For certain picornaviruses, within the enterovirus genus, interactions between the virus capsid proteins and the 2C non-structural protein have been implicated in the process of virus morphogenesis [14–16].

## Results

### Modification of the VP1/2A cleavage site in a serotype A FMDV

In order to determine whether the key elements of the results obtained with the serotype O FMDV sequences [13] also applied to serotype A FMDV, the effect of modifying the VP1/2A cleavage site within a serotype A FMDV was examined. The VP1/2A cleavage site sequence in the A22 strain of FMDV (AP**A**KQ/LLNFD) differs at just one out of the 10 residues flanking the junction from the serotype O (strain O1 Manisa, abbreviated throughout as O1M) sequence (AP**V**KQ/LLNFD) analysed previously [6, 13] (see Fig. 1). The K210E substitution (at the P2 position) was introduced into a full-length FMDV cDNA, based on the backbone of a chimeric O1 Kaufbeuren (O1K) virus containing the coding sequence for the VP2-VP3-VP1-2A region of the A22 capsid protein precursor (Fig. 1). Virus was successfully rescued from this chimeric wt O1K/A22 plasmid and also from the O1K/A22 VP1 K210E mutant (this had changed the codon encoding VP1 residue 210 from AAA to GAA). However, when the capsid protein coding sequences within the rescued virus were determined, it was found that the K210E substitution in VP1 had reverted in the virus by passage 2 (Psg 2) to the wt sequence (this only requires a single nt change) (see Fig. 1).

Two further modifications were, therefore, introduced into the serotype A viruses: the 2A L2P modification was made in isolation (using 3 nt changes, TTG to CCA) and a double mutant containing both the K210E substitution (the single nt change) and the 2A L2P substitution. Viruses were rescued successfully from both of these mutant plasmids. Consensus sequencing indicated that the expected mutations were still present within these rescued viruses and that no other mutations were detected within the VP2-2A coding region (see Fig. 1).

**Fig. 2.**
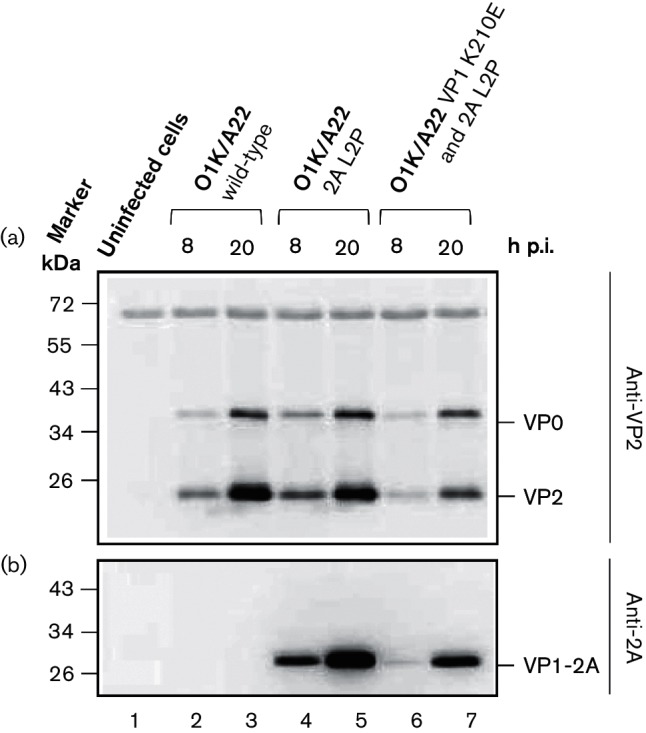
Detection of FMDV capsid proteins in BHK cells infected with O1K/A22 wt and mutant viruses. Baby hamster kidney (BHK) cells were infected with O1K/A22 wt or mutant viruses (single mutant 2A L2P or double mutant VP1 K210E and 2A L2P) (m.o.i. of 0.1), and whole-cell lysates were prepared at the indicated times post-infection (p.i.). The presence of FMDV VP2 (and its precursor VP0) was detected by immunoblotting using anti-VP2 antibodies (a) and FMDV 2A (attached to VP1 as VP1-2A) was detected using anti-2A antibodies (b). Uninfected BHK cells were used as a negative control. Molecular mass markers (kDa) are indicated on the left.

Analysis of the FMDV capsid proteins within cells infected with the wt and mutant O1K/A22 viruses, as determined by immunoblotting using anti-VP2 and anti-2A antibodies, is shown in Fig. 2. As expected, the production of VP0 and VP2 was very similar in each of the infected cell extracts (Fig. 2a, lanes 2–7). However, the presence of the uncleaved VP1-2A was only observed with the mutant viruses, either containing the 2A L2P substitution alone (Fig. 2b, lanes 4 and 5) or with the double mutant (VP1 K210E and 2A L2P) (Fig. 2b, lanes 6 and 7).

**Fig. 3.**
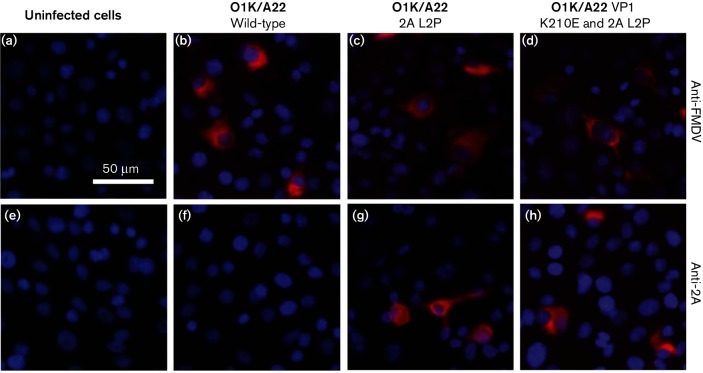
IF staining of FMDV proteins within serotype A FMDV-infected cells. FMDV proteins within uninfected or FMDV-infected cells (using m.o.i. of 0.1) were detected (at 8 h p.i.) using an anti-FMDV A-Iraq polyclonal antibody (a–d) or an anti-2A antibody (e–h) and a secondary antibody labelled with Alexa Fluor 568 (red). Uninfected cells are shown in (a) and (e). Cells were infected with the viruses O1K/A22 (wt) (b and f), O1K/A22 2A L2P (c and g) or O1K/A22 VP1 K210E and 2A L2P (d and h) as indicated. The cellular nuclei were visualized with DAPI (blue). Bar, 50 µm.

These results were confirmed using immunofluorescence (IF) studies (Fig. 3). Consistent with the immunoblotting data, the presence of FMDV 2A (still attached to VP1) was detected in cells infected with the O1K/A22 2A L2P mutant virus (Fig. 3g) and with the double mutant (O1K/A22 VP1 K210E and 2A L2P) virus (Fig. 3h). In contrast, no signal for the 2A peptide was observed in cells infected with the wt O1K/A22 virus (Fig. 3f) or in uninfected cells (Fig. 3e). The presence of the FMDV capsid proteins could be detected in cells infected with each of the viruses (Fig. 3b–d) but not in uninfected cells (Fig. 3a). These results are consistent with those obtained using the serotype O FMDVs previously [13]. It seems that the free 2A peptide is not efficiently detected within cells using the IF approach; it is assumed that either it breaks down very quickly or the conditions of the IF assay are not suitable for detection of this short peptide.

### Use of next-generation sequencing to determine sequence diversity within rescued FMDVs

As described previously [6, 13], consensus sequencing of the capsid coding region (P1-2A) of the serotype O FMDVs rescued from the K210E and E83K mutant forms of the O1K/O1M cDNAs identified the presence of additional amino acid substitutions in the rescued viruses. We wished to analyse these adaptations in more detail, in particular to examine the appearance of sub-consensus level changes throughout the near-complete genome sequence including the complete polyprotein coding region. To achieve this, RNA was extracted from the rescued O1K/O1M VP1 E83K and O1K/O1M VP1 K210E viruses (as described by Gullberg *et al.* [6, 13]) at Psg 2 and/or Psg 3. Two separate, but overlapping, cDNA fragments including the near-complete genome [ca. 8 kb, downstream of the poly(C) tract] were produced by reverse transcription (RT)-PCR, mixed and then sequenced using next-generation sequencing (NGS) at a coverage of about 5000 reads per nt (except near the extreme 5′- and 3′-termini).

This analysis showed that the rescued virus O1K/O1M VP1 E83K retained the expected substitution (encoding E83K) in 100 % of the progeny at Psg 2, but there was some heterogeneity in the sequence near the VP1/2A junction (see Table 1). As described earlier, the consensus sequencing indicated that a substitution (L2P) within the 2A sequence occurred within this rescued virus [13]. The analysis by NGS (see Table 1) demonstrated that at Psg 2, some 83 % of the reads corresponded to this L2P substitution in 2A, while two other variants were also present (albeit at relatively low levels, 4 and 9 %), which each encoded the K210N substitution in VP1 (c.f. the K210E change described previously in O1M, [13]). No other coding changes were present anywhere in the genome at an abundance of more than 3 %.

Consensus sequence analysis of the rescued O1K/O1M VP1 K210E virus has shown previously the generation of the E83K substitution [6]. Using NGS, it was found that the E83K substitution in VP1 was encoded in 78 % of the reads generated at Psg 2 and in 99 % of the reads at Psg 3 (Table 1), consistent with the earlier consensus sequencing [6]. The K210E substitution was maintained in this rescued virus in 100 % of the reads at Psg 2 and Psg 3.

**Table 1.** Analysis of SNVs within rescued O1K/O1M viruses as determined by NGS

Nt position	Wt	Variant	O1K/O1M VP1 E83K (Psg 2) (%)∗	O1K/O1M VP1 K210E (Psg 2) (%)∗	O1K/O1M VP1 K210E (Psg 3) (%)∗	SNV effect (whole polyprotein)	SNV effect (individual protein)
762	T	C	6	–	–	(in 5′ UTR)	–
948	T	G	–	1	3	(in 5′ UTR)	–
1811	T	C	6	–	–	–	–
1875	A	G	3	–	–	T253A	VP4 (T52A)
2102	C	T	3	–	–	–	–
2357	A	G	–	3	3	–	–
3537	G	A	100	78	99	E807K	VP1 (E83K)
3918	A	G	–	100	100	K934E	VP1 (K210E)
3920	A	C	4	–	–	K934N	VP1 (K210N)
3920	A	T	9	–	–	K934N	VP1 (K210N)
3928	T	C	83	–	–	L937P	2A (L2P)
5166	A	G	3	–	–	S1350G	2C (S243G)
7392	T	C	–	5	7	F2092L	3D (F228L)
7765	A	G	2	–	–	K2216R	3D (K353R)

SNV, single nucleotide variant.

∗The percentage of each variant (≥1 %) is given to the nearest integer.

For the rescued serotype A viruses, consensus (Sanger) sequencing of the rescued O1K/A22 2A L2P virus demonstrated the maintenance of the introduced changes and did not reveal any additional modifications, resulting in amino acid substitutions within the P1-2A coding region. This was confirmed by NGS, but an A-to-G nucleotide change, resulting in a single amino acid substitution (T44 to A within the 2C protein), was found in 13 % of the reads from the rescued virus (Table 2). Within the O1K/A22 VP1 K210E and 2A L2P virus, only the expected changes in the P1-2A coding region were observed, but an additional change, resulting in the amino acid substitution A73V in the 2C protein, was detected in 100 % of the sequence reads (see Table 2). The plasmid pO1K/A22 K210E L2P from which this virus was rescued has been confirmed as having the expected sequence in the 2C coding region (100 % identity to O1K, data not shown), and thus, this sequence change had occurred during virus rescue. The significance of these changes in 2C is unknown; neither residue is completely conserved among FMDV strains [17]. The A73V change in 2C was not present in the rescued O1K/A22 wt or the O1K/A22 2AL2P, and there was no evidence for the T44A amino acid substitution in either the O1K/A22 wt or the O1K/A22 VP1 K210E and 2A L2P virus that was encoded in a minority of the O1K/A22 2A L2P virus reads.

**Table 2.** Analysis of SNVs within rescued O1K/A22 viruses as determined by NGS

**Nt position**	**Wt**	**Variant**	**O1K/A22 (wt)** **(Psg 2) (%)∗**	**O1K/A22 2A L2P** **(Psg** **3) (%)**∗	**O1K/A22 VP1** **K210E 2A L2P** **(Psg 3) (%)**∗			**SNV effect (whole polyprotein)**	**SNV effect (individual protein)**
932	A	C	–	2		–	(in 5′-UTR)		–
1060	T	G	2	–		–	(in 5′-UTR)		–
1124	T	A	–	7		–	N2K		L (N2K)
1341	C	T	–	–		4	P75S		L (P75S)
1400	G	T	–	–		2	–		–
1865	C	T	–	2		–	–		–
2175	T	C	–	2		–	F353L		VP2 (F67L)
2342	A	G	–	–		2	–		–
2684	C	T	–	3		–	–		–
3154	T	C	–	1		–	V679A		VP3 (V174A)
3824	G	C	2	–		–	–		–
3884	T	C	–	8		–	–		–
3921	A	G	–	–		100	K935E		VP1 (K210E)
3930	T	C	–	90		100	†		†
3931	T	C	–	98		100	L938P†		2A (L2P)†
3932	G	A	–	99		100	†		†
4572	A	G	–	13		–	T1152A		2C (T44A)
4660	C	T	–	–		100	A1181V		2C (A73V)
4821	A	C	–	1		–	I1235L		2C (I127L)
4843	G	T	–	1		–	R1242I		2C (R134I)
5080	C	T	–	5		–	T1321I		2C (T213I)
6924	C	T	–	11		–	–		–
7170	T	C	3	–		–	Y2018H		3D (Y155H)
7358	C	T	–	–		4	–		–

SNV, single nucleotide variant.

∗The percentage of each variant (≥1 %) is given to the nearest integer.

†Change of the TTG to CCA codon was introduced by site-directed mutagenesis, and the three changes together result in the 2A (L2P) substitution.

### Exploring potential sequence diversity at the VP1/2A cleavage site

The results described previously [6, 13] indicated that modification of the VP1/2A junction sequence from PxKQ/xLNF at the P2 position (from K to E) or the P2′ position (from L to P) was sufficient to strongly inhibit cleavage by 3C^pro^ at this protein junction, but these changes did not affect virus viability. Furthermore, the NGS data (Table 1) indicated that the K-to-N substitution at residue 210 of VP1 was also probably viable since some 13 % of the virus population acquired this change. To establish the range of amino acid substitutions that could be tolerated at this junction, mutagenesis of the codon for residue 210 within VP1 was undertaken within the context of the O1K/A22 full-length cDNA. The mutagenesis generated nine different codon sequences that encoded seven distinct amino acid substitutions (see Table 3). RNA transcripts were produced from the mutant cDNAs and electroporated into baby hamster kidney (BHK) cells, and infectious viruses were rescued in each case. From the virus harvests, RNA was extracted and the sequence of VP1-2A coding region was determined, and changes (if any) are shown in Table 3. Consistent with the studies described above, the O1K/A22 VP1 K210E mutant (with a single nt change, GAA) again reverted to the parental sequence; however, when 2 nt changes were introduced (GAG) to produce the K210E substitution, then viruses that retained these 2 nt changes were obtained. Thus, the VP1 substitutions K210Q, K210R, K210A, K210V, K210M, K210N and K210E (as the GAG double mutant) were all viable without reversion or other adaptation within VP1 (Table 3). Using consensus-level sequencing, no changes in the 2C coding region were detected in any of these rescued viruses either (data not shown).

**Table 3.** Influence of residue K210 in VP1 on VP1/2A junction cleavage in FMDV-infected cells

**Virus**	**VP1 210 codon**	**Viability**	**Rescued virus sequence**	**Rescued virus amino acid**	**Comment**	**VP1/2A cleavage**∗
pO1K/A22 K210	AAA	+	AAA	K	wt	**+**
pO1K/A22 K210Q	**C**AA‡	+	CAA	Q		−
pO1K/A22 K210R	A**G**A‡	+	AGA	R		+
pO1K/A22 K210A(v1)**†**	**GC**A‡	+	GCA	A		−
pO1K/A22 K210A(v2)**†**	**GCG‡**	+	GCG	A		−
pO1K/A22 K210V	**GTT‡**	+	GTT	V		−
pO1K/A22 K210M	A**TG‡**	+	ATG	M		−
pO1K/A22 K210N	AA**C‡**	+	AAC	N		−
pO1K/A22 K210E(v1)**†**	**G**AA‡	+	AAA	K	Reversion	**+**
pO1K/A22 K210E(v2)**†**	**G**A**G‡**	+	GAG	E		−

**Fig. 4.**
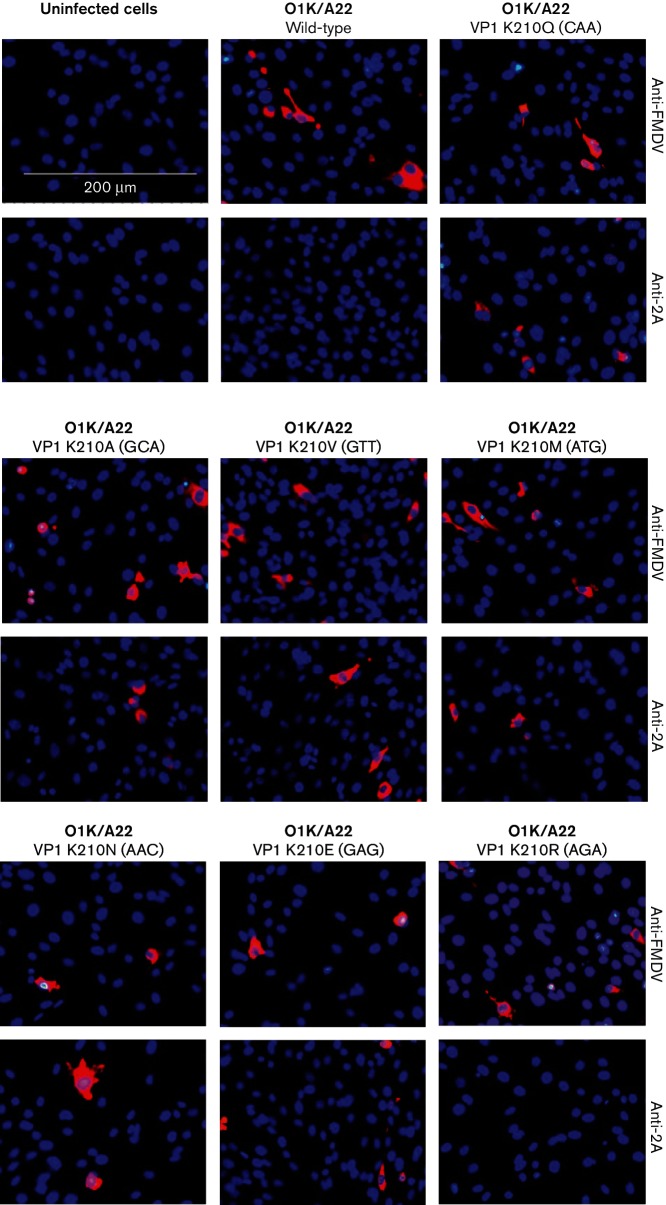
Determination of VP1/2A cleavage by IF staining for FMDV proteins within cells. FMDV proteins within uninfected or FMDV-infected cells (using m.o.i. of 0.1) were detected (at 6 h p.i.) using an anti-FMDV A-Iraq polyclonal antibody or an anti-2A antibody (as indicated) and a secondary antibody labelled with Alexa Fluor 568 (red) as in Fig. 3. The codon for residue 210 within VP1 (in parentheses) and the resulting individual amino acid residue within the different rescued viruses are indicated. Uninfected cells were used as a negative control. The cellular nuclei were visualized with DAPI (blue). Bar, 200 µm.

**Fig. 5.**
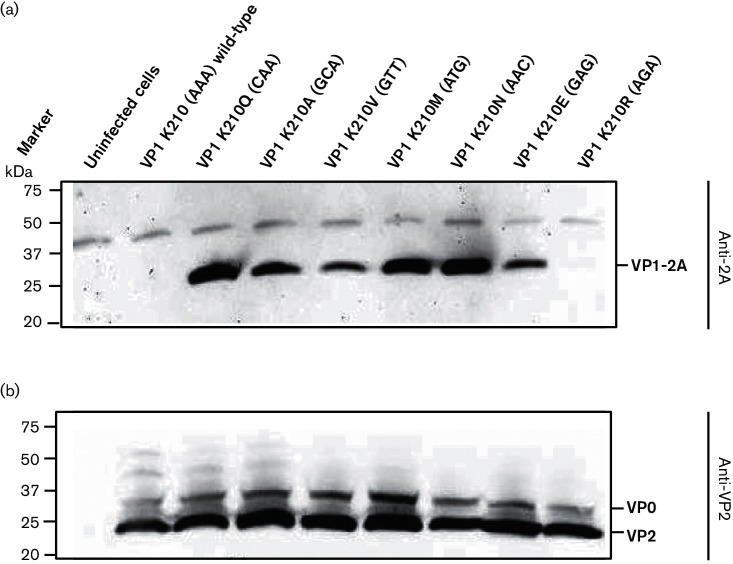
Assessment of FMDV VP1/2A cleavage in FMDV-infected BHK cells by immunoblotting. Uninfected or FMDV-infected BHK cell lysates were analysed by SDS-PAGE and immunoblotting (as in Fig. 2). Where applicable, the cells were infected with the indicated viruses (at an m.o.i. of 0.1) and the presence of FMDV 2A (attached to VP1 as VP1-2A) was detected using anti-2A antibodies (a), while FMDV VP2 (and its precursor VP0) was detected by immunoblotting using anti-VP2 antibodies (b). Uninfected cells were used as a negative control. Molecular mass markers (kDa) are indicated on the left.

**∗**VP1/2A cleavage was assessed from IF staining using anti-2A antibodies (as in Fig. 4) and by immunoblotting (Fig. 5a).

**†**v1 or v2 to distinguish different codons.

**‡**Nucleotide changes in this codon are indicated in bold font.

The cleavage of the VP1/2A junction in cells infected with the rescued mutant viruses was assessed using the IF assay, as described above. The presence of the FMDV capsid proteins (using anti-FMDV antisera) and of the VP1-2A (using anti-2A antibodies) was determined in BHK cells infected with the different viruses. FMDV infection, but with no staining for VP1-2A, was observed in cells infected with the viruses containing the VP1 residue K210 (wt) and R210 (Fig. 4). In contrast, cells infected with the rescued viruses having the substitutions in VP1 K210Q, K210A, K210V, K210M, K210N and K210E (double mutant) each showed staining both for the FMDV capsid proteins and for 2A (using the anti-2A antisera), indicative of blocked VP1/2A cleavage (see Fig. 4, Table 3). No staining with either antiserum was observed in uninfected cells, as expected (Fig. 4).

To confirm these results, immunoblotting was performed using anti-2A antibodies to determine the presence of the uncleaved VP1-2A within infected cells. The results are shown in Fig. 5(a). Consistent with the IF results, no VP1-2A product was detected in uninfected cells or in wt (K210) or mutant K210R FMDV-infected cells. In contrast, the presence of VP1-2A was readily apparent within cells infected with mutant FMDVs having the K210Q, K210A, K210V, K210M, K210N and K210E substitutions. The presence of FMDV capsid proteins in the lysates from cells infected with each of the different FMDV variants was verified using anti-VP2 antibodies that recognize both VP0 and VP2 (Fig. 5b). These results support the IF data and indicate that the VP1/2A junction is only cleaved when residue 210 in VP1 is either K or R (these are both basic residues).

## Discussion

In our earlier studies, it was shown that the K210E substitution in VP1 within the FMDV O1M capsid inhibited cleavage of the VP1/2A junction and resulted in generation of a second site substitution (E83K in VP1) in the mutant virus [6]. Furthermore, introduction of the E83K substitution alone in VP1 resulted in the production of another second site change (with a substitution of L2P in 2A) that also blocked VP1/2A junction processing in cells infected with the rescued virus [13]. In contrast, this study has shown that when the K210E substitution (GAA mutant) was introduced into the VP1 of a serotype A virus (O1K/A22), the virus reverted to wt (AAA) very rapidly (single nt change). However, introduction of 2 nt changes (GAG codon) enabled the K210E substitution to be maintained. Consistent with the serotype O virus results [6, 13], this amino acid substitution alone was sufficient to block VP1/2A cleavage (see Figs 4 and 5). In addition, introduction of the 2A L2P change alone (employing 3 nt changes) or also with the K210E substitution resulted in the generation of viruses which maintained each of these changes. Furthermore, the presence of the uncleaved VP1-2A protein was observed within cells infected with these serotype A viruses (Figs 2 and 3). Thus, consistent with results using the O1K/O1M FMDV [6, 13], it is possible to obtain infectious serotype A FMDVs, containing the uncleaved VP1-2A. However, in contrast to the results using the serotype O virus, there was no apparent selection for a substitution at residue E83 in VP1 (or elsewhere within the VP1) within the serotype A background. The basis for this difference is not known, but it is noteworthy that the serotype A capsid proteins assemble into FMDV empty capsids much more efficiently than the serotype O proteins [18, 19]. It is also apparent that some of the rescued serotype A viruses, with the VP1/2A junction rendered non-cleavable, acquired second site mutations within the non-structural protein 2C (see Table 2). In particular, the A73V variant within 2C was encoded by 100 % of the sequence reads at Psg 3 of the rescued O1K/A22 VP1-K210E and 2A-L2P virus. This suggests a strong selective pressure for this amino acid substitution. The significance of this is currently unknown, but there is some evidence for interactions between the capsid proteins and the 2C protein of enteroviruses (e.g. poliovirus) during virus morphogenesis [14–16].

Interestingly, it was also observed, using NGS, that during the rescue of the O1K/O1M VP1 E83K virus, a minority of the virus population encoded a K210N substitution in VP1 (c.f. the K210E substitution observed in a laboratory grown O1M virus, [6]). It should be noted that the majority of the serotype O VP1 E83K mutant virus RNA acquired the 2A L2P substitution during the virus rescue procedure (see Table 1). This encouraged analysis of the range of substitutions that can be accommodated at residue 210 in VP1; this is at position P2 relative to the VP1/2A cleavage site. Within the serotype A background, there appears to be significant selection pressure against the K210E substitution, since following independent transfections of a mutant RNA containing a single nt change (GAA) reversion to the wt (AAA) sequence occurred. However, when 2 nt changes were used (GAG) to make this amino acid substitution, then the K210E substitution was maintained. The single nt substitution to make the K210E substitution was also maintained in the O1K/A22 K210E and L2P double mutant; presumably the presence of the two substitutions that blocked VP1/2A cleavage overcame the selective pressure for reversion. In addition, a range of other amino acid substitutions was tolerated. Most of these substitutions (e.g. K210A, K210V, K210M and K210N) blocked cleavage of the VP1/2A junction; thus, the VP1-2A product was stable (see Figs 4 and 5). In contrast, the 2A peptide was released from the VP1 in the K210R mutant. Therefore, it appears that the presence of a positively charged residue (K or R) at residue 210 in VP1 is essential for VP1/2A cleavage, and a negative charge (as in K210E) is less well tolerated by itself and was selected against. These results are consistent with the strong predominance of the K and R residues at this position in the ‘logos plot’ generated by Curry *et al*. [11] for 3C^pro^ cleavage sites, with Q at the P1 position, based on the known sequences of over 100 strains of FMDV.

Using peptide cleavage assays, the VP1/2A peptide was the most efficiently cleaved substrate for FMDV 3C^pro^ [10]. However, making the K210A substitution abrogated cleavage of this peptide, in accordance with the results presented here. Zunszain *et al.* [12] have described additional modifications to the peptide substrate, which corresponds to the VP1/2A junction. Changing the P2 residue in the cleavage site (corresponding to K210 in VP1) from K to R or to ornithine (also positively charged) had relatively modest effects on the cleavage rate. However, substitution to the neutral T residue abrogated cleavage as observed here, with the K210 changed to Q, A, V, M or N (see Table 3, Figs 4 and 5).

It has been proposed that the FMDV 3C^pro^ may be a good target for the development of an antiviral agent [20]. Furthermore, it was shown that compounds that resemble the peptide substrate can act as an efficient inhibitor of this protease. The presence of a positively charged residue at the P2 position generated the most effective inhibitors, while compounds containing neutral residues (e.g. G and Q) or a negatively charged residue (E) at this position were much less effective, consistent with the poor cleavage of the VP1/2A junction seen here in viruses containing equivalent substitutions.

It is important to note that viable FMDVs with the VP1/2A junction uncleaved can be obtained (as here, and as described previously for serotype O viruses [6, 13]). Thus, it may be wise to focus screens for such antiviral agents on other 3C^pro^ cleavage sites so that the block on polyprotein processing is most effective at inhibiting virus replication.

The results presented here demonstrate that the combination of reverse genetics and NGS provides powerful tools to direct and identify virus adaptation, thus permitting novel aspects of the virus biology to be identified.

## Methods

### Plasmid construction

The structures of plasmids containing full-length FMDV cDNAs used in this study are indicated in Fig. 1. The chimeric pO1K/A22 plasmids (containing the A22 Iraq capsid coding sequences, as used in [19, 21]) in the FMDV O1K backbone were generated using the same procedures as used previously for the production of the pO1K/O1M VP1 K210E [6]. Briefly, the cDNAs corresponding to the A22 VP2-2A coding region were amplified from pGEM-3Z-A-P1-2A-mIRES-3C [5] and pGEM-3Z-A-P1-2A-mIRES-3C VP1K210E [6] with primers FMDVA_*Nhe*IVP4VP2_Fw and FMDVA_*Apa*I2A2B_Re (see Table S1, available in the online Supplementary Material) and used to generate the full-length cDNAs termed pO1K/A22 wt and pO1K/A22 VP1 K210E, respectively (Fig. 1). In order to produce pO1K/A22 2A L2P and the double mutant pO1K/A22 VP1 K210E and 2A L2P, intermediate plasmids (using pO1K/A22 wt and pO1K/A22 VP1K210E, as described above, as templates) were generated. This was achieved using the QuikChange site-directed mutagenesis method (with *Pfu*Turbo DNA polymerase; Stratagene), according to the manufacturer’s instructions, with primers containing the desired modifications (see Table S1, namely FMDVA_2AL2P_Fw together with FMDVA_2AL2P_Re or FMDVA_VP1K210E_2AL2P_Re). The subsequent steps to produce the four pO1K/A22 variants were performed essentially as described for the serotype O plasmid pO1K/O1M VP1 E83K [13]. Plasmids were amplified in *Escherichia coli* Top10 cells (Invitrogen), purified (Midiprep kit; Thermo Scientific) and verified by sequencing of the capsid coding region (and for pO1K/A22 VP1 K210E the 2C coding region as well) with a BigDye Terminator v. 3.1 Cycle Sequencing kit and a 3500 Genetic Analyzer (Applied Biosystems).

Additional mutations, encoding changes at the VP1/2A junction, were introduced into the pO1K/A22 full-length FMDV cDNA (Fig. 1) by site-directed mutagenesis using a megaprimer (146 bp) that was prepared by PCR using primer O1PN20 and primer 13LPN21 (see Table S1) that had NNN at the position corresponding to the codon for the VP1 residue 210. This degenerate megaprimer was used, with the pO1K/A22 full-length cDNA template and *Pfu*Turbo DNA polymerase (as above) to generate a collection of nine plasmids encoding a variety of different amino acids, with diverse properties, in place of VP1 residue K210; this is at position P2 relative to the VP1/2A cleavage site. The details of each modification made are indicated in Table 3.

### Rescue of modified viruses from cDNA

Plasmids pO1K/A22 (wt), pO1K/A22 VP1 210E, pO1K/A22 2A L2P and pO1K/A22 VP1 K210E 2A L2P (as shown in Fig. 1) containing the full-length FMDV cDNA sequences were linearized by digestion with *Hpa*I, purified (using a QIAquick PCR purification kit; Qiagen) and transcribed *in vitro* using T7 RNA polymerase (Megascript kit; Ambion). The transcripts were analysed using agarose gel electrophoresis and then introduced into BHK cells by electroporation as described previously [22, 23]. At 2 days post-electroporation, the rescued viruses were harvested and amplified in one, or two, subsequent passages (Psg 2 and Psg 3) in BHK cells. After these passages, viral RNA was extracted (QIAamp RNA Blood Mini kit; Qiagen) and reverse transcribed using Ready-To-Go You-Prime First-Strand Beads (GE Healthcare Life Sciences), and the FMDV cDNA corresponding to the VP2-2A coding region was amplified in a PCR (Expand High-Fidelity PCR System; Roche). Control reactions, lacking reverse transcriptase, were used to show that the PCR products obtained were derived from the viral RNA and not from residual DNA template. The amplicons (~2000 bp) including the entire VP2-2A coding region were visualized in agarose gels, purified (GeneJET Gel Extraction kit, Thermo Scientific) and sequenced, on both strands, as above. Sequences were analysed using Vector NTI software (Invitrogen).

For the library of VP1 K210 mutants, mutant viruses were rescued essentially as described above. The sequencing covered the VP1-2A coding region (from a PCR product of ca 700 bp) both before and after virus rescue. The sequence of the 2C coding region was also determined in selected cases. The rescued viruses were titrated (and were in most cases ca 10^6^ to 10^7^ TCID_50_ ml^−1^), and in some cases, a fourth passage in BHK cells was required to reach this titre.

The rescued serotype O viruses O1K/O1M VP1 E83K and O1K/O1M VP1 K210E viruses have been described previously [6, 13].

### RT-PCR and NGS

For the purpose of NGS, extracted RNA (isolated as described above from virus harvests) was converted to cDNA using SuperScript III (Invitrogen) with a T_27_ reverse primer according to the manufacturer’s protocol. Two cDNA amplicons were prepared by PCR using Phusion DNA polymerase (Thermo Fisher) with the primers 13-N PN 2+10-P PN 30 (Table S1) and separately 8-A PN 200+13-N PN 3 (Table S1). These overlapping fragments (ca. 4 and 4.2 kb, respectively) correspond to most of the FMDV genome [downstream of the poly(C) tract, see Belsham [1]], including the complete polyprotein coding region (ca. 7 kb) but excluding the S-fragment at the 5′-terminus of the viral genome. The fragments were gel purified, mixed and then analysed by NGS essentially as described previously [24, 25]. The parental sequences of the FMDV chimeric O1K/O1M cDNA (as described by Gullberg *et al.* [6]) were assembled from the O1K sequence (accession no. X00871) and the coding sequence for the O1M capsid proteins (from accession no. AY593823) with known differences (see [6]), while the chimeric O1K/A22 sequence was generated using the O1K sequence and the A22 Iraq sequence ([17], accession no. AY593764.1). The derived sequences were used as the reference for mapping of sequence reads using SAMtools [26], VarScan 2 [27] and VCFtools [28], in succession, in order to generate consensus sequences from the mapped reads. Subsequently, consensus sequences were aligned using MAFFT in Geneious (Biomatters). Finally, a combination of SAMtools [26], Lo-Freq [29] and SnpEff [30] was used for downstream single nucleotide variant (SNV) analysis.

### Virus infection of BHK cells

Virus titres were determined, as tissue culture infectious doses (TCID_50_), by titration in BHK cells according to standard procedures [31].

Monolayers of BHK cells, grown in 35 mm wells, were inoculated with the rescued viruses at a m.o.i. of 0.1 TCID_50_/cell. At the indicated times post-infection (p.i.), cell lysates were prepared using 20 mM Tris/HCl (pH 8.0), 125 mM NaCl and 0.5 % NP-40, and clarified by centrifugation at 18 000 ***g*** for 10 min at 4 °C.

### Immunoblot analysis

Immunoblotting was performed, using cell lysates, according to standard methods as described previously [21]. Briefly, aliquots of cell lysate were mixed with Laemmli sample buffer (with 25 mM DTT), and the proteins were separated by SDS-PAGE (12.5 or 4–15 % polyacrylamide) and transferred to PVDF membranes (Millipore). Specific proteins were detected with primary antibodies recognizing FMDV VP2 (monoclonal antibody 4B2, a gift from L. Yu, as described by Yu *et al.* [32]) and FMDV 2A (ABS31; Millipore). Bound proteins were visualized using appropriate HRP-conjugated secondary antibodies (Dako) and a chemiluminescence detection kit (ECL Prime, Amersham) with a Chemi-Doc XRS system (Bio-Rad).

### IF assays

Monolayers of BHK cells, grown on glass coverslips in 35 mm well plates, were infected with the rescued O1K/A22 viruses (m.o.i. of 0.1). At 6–8 h p.i., the cells were fixed, stained and mounted as previously described [6] using rabbit anti-FMDV A-Iraq serum and anti-FMDV 2A (ABS31, as above) followed by a donkey Alexa Fluor 568-labelled anti-rabbit IgG (A10042, Life Technologies). The slides were mounted with Vectashield (Vector Laboratories) containing DAPI, and images were captured using an epifluorescence microscope.

## Supplementary Data

30 Supplementary File 1
