# Supplementary material for: Determinants of the VP1/2A junction cleavage by the 3C protease in foot-and-mouth disease virus-infected cells
Source: J Gen Virol. 2017 Apr 1;98(3):385–95. doi: 10.1099/jgv.0.000664 (PMC5797948; doi:10.1099/jgv.0.000664)
Supplement: Supplementary File 1 [file jgv-98-385-s001.pdf]

Supplementary Table S1. Primers used for plasmid construction and cDNA synthesis

Primer name	Sequence (5´-3´)
FMDVA_*Nhe*IVP4VP2_Fw	CGCTCTGCTAGCCGATAAGAAGACCGAGGAGACCA
FMDVA_*Apa*I2A2B_Re	CTACTAGGGCCCGGGGTTGGACTCAACGTCTCCTG
FMDVA_2AL2P_Fw	CAACTT***CCA***AACTTCGATTTGCTCAAGTTGGCAGGAGAC
FMDVA_2AL2P_Re	GAAGTT***TGG***AAGTTGTTTTGCAGGTGCAATGATCTTCTG
FMDVA_VP1K210E_2AL2P_Re	GAAGTT***TGG***AAGTTGTT**C**TGCAGGTGCAATGATCTTCTG
13-N PN2	AAGTTTTACCGTCGTTCCCGACGTAAAAGGGAGGTAACCAC AAGCTTGAA
10-P PN 30	TCTGGACAGCACCTTTGTCG
8-A PN 200	GAGACGTTGAGTCCAACCC
13-N PN 3	CCGTAGGAGTGAAAATCCCGAAAGGGTTTTTCCCGCTTCCTT AATCCAAA
O1PN20	GACATGTCCTCCTGCATCTG
13LPN21	GCACCTGCA***NNN***CAACTTTTGAAC

[a] Underlined sequences represent restriction enzyme sites *NheI* and *ApaI*. Nucleotide changes producing amino acids substitutions are shown in boldface italics.
